# Role of human *HGFIN/nmb *in breast cancer

**DOI:** 10.1186/bcr1764

**Published:** 2007-09-10

**Authors:** Rebecca L Metz, Prem S Patel, Meera Hameed, Margaret Bryan, Pranela Rameshwar

**Affiliations:** 1 Department of Medicine, UMDNJ-New Jersey Medical School, Newark, NJ, USA; 2 Department of Obstetrics, Gynecology and Women's Health, UMDNJ-New Jersey Medical School, Newark, NJ, USA; 3 Brookdale University Hospital and Medical Center, Division of Trauma, Brooklyn, NY, USA; 4 Department of Laboratory Medicine and Pathology, UMDNJ-New Jersey Medical School, Newark, NJ, USA

## Abstract

**Introduction:**

HGFIN, previously identified as nmb, and its homolog osteoactivin are single transmembrane proteins that are expressed in differentiated immune cells. These proteins exhibit properties that could potentiate tumorigenesis or decrease invasiveness. These seemingly opposing roles of HGFIN suggest that this protein might be central to malignancies and might also behave as a tumor suppressor. Consistent with the reported roles for HGFIN is the fact that this gene is regulated by p53 through multiple binding sites in the 5' flanking region, and is expressed in osteoblasts.

**Methods:**

This study used siRNA to knock-out *HGFIN *in non-tumorigenic breast cells and ectopically expressed HGFIN in breast cancer cells. In addition, *in situ *hybridization studies analyzed primary breast tissues from archived breast surgeries. Reporter gene assays studied the untranslated exon 1 of *HGFIN*.

**Results:**

HGFIN expression led to reduced cell growth of breast cancer cells and reduced migration. At the molecular level, reporter gene analyses determined the untranslated exon 1 to be a negative regulator of the upstream enhancing effect. Ectopic expression of wild-type p53 in breast cancer cells that expressed endogenous mutant p53 resulted in increased *HGFIN *reporter gene activities.

**Conclusion:**

As the majority of cancer cells have mutations in p53, further studies on the relationship between p53 and HGFIN expression, and its role in tumor genesis and bone invasion, might uncover novel therapy targets for breast and other cancers. The results show a central role for p53 in HGFIN expression, which appears to determine the behavior of the cancer cells.

## Introduction

Hematopoietic growth factor inducible neurokinin-1 type (HGFIN; also known as nmb) is a single transmembrane protein located in human chromosome 7 [1,2]. HGFIN shares sequence similarities with the G-protein, 7-transmembrane coupled neurokinin-1 (NK1) receptor [1]. This similarity results in HGFIN being able to interact with the high affinity ligand for NK1, substance P [1]. The murine homolog of HGFIN, osteoactivin, has been reported to upregulate matrix metalloproteinase-3 and -9 in atrophied skeletal muscles from denervation [3]. Osteoacvtivin is involved in osteoblast development and function [2,4,5]. The fact that osteoactivin is expressed in differentiated osteoblasts is consistent with human HGFIN being linked to differentiated immune cells [1,6]. HGFIN has been reported to act as a negative regulator of inflammation [7,8]. A recent description of a protein with 99% homology to HGFIN, DC-HIL show that this gene is promotes adhesion in an RGD-dependent manner [9].

Osteoactivin and HGFIN are widely expressed in normal and malignant cells [10]. Osteoactivin is expressed in breast cancer cells and has been associated with bone invasion, an aggressive form of the disease [11]. *HGFIN *expression is partly regulated by p53 through multiple sites within the 5' flanking region [12]. In contrast to its expression in cancer cells, in non-transformed cells *HGFIN *expression appears to be critical in cell cycle quiescence [1,12]. The link between p53 and the regulation of *HGFIN *expression leaves the question of the role of *HGFIN *in tumor development open. Presence of *HGFIN *confers low metastatic potential in melanoma cells [2].

This study further investigates the regulation of *HGFIN*, and also determines its involvement in breast cancer. Here, we report on a repressive effect of the non-coding exon 1. We also report on the cause–effect relationship between defect in *HGFIN *and transformation of non-tumorigenic breast cells.

## Materials and methods

### Reagents and antibodies

RPMI-1640, DMEM, α-MEM, alkaline phosphatase-conjugated goat anti-rabbit IgG, neurokinin-A, and anti-Flag were purchased from Sigma (St Louis, MO, USA). Fetal calf serum (FCS) and horse serum were purchased from Hyclone Laboratories (Logan, UT, USA).

### Cell lines

K562, MCF12A (non-tumorigenic), MCF10 (non-tumorigenic), DU4475 (carcinoma), HCC70 and T47D (low invasive) were purchased from American Type Culture Collection (ATCC; Manassas, VA, USA) and cultured according to their instructions. The highly aggressive cancer cell line MDA-MB-231 was obtained from Dr Ian Whitehead, New Jersey Medical School (Newark, NJ, USA), and was originally described by ATCC. CCL64 has been described previously [13].

### Vectors and reporter gene assay

pGL3-basic and the luciferase detection kit were purchased from Promega (Madison, WI, USA). The β-galactosidase (β-gal) detection kit and pHyg were purchased from Clontech (Palo Alto, CA, USA). pFLAG-CMV2-HGFIN and pPMSKH1 (siRNA) were as previously described [1,14]. pCR2.1 was purchased from Invitrogen (Carlsbad, CA, USA). The p53 expression vectors and mutants were kindly provided by Dr Yuzuru Shiio (Institute for Systems Biology, Seattle, WA, USA): pME18S-SN3 wild-type human p53, pME18S-SCX3 contained 143 Val→Ala mutant human p53, and pPME18S [15]. The vectors are under the SRα promoter. The expression vectors encode both the N- and C-termini of p53 [15].

### Cloning HGFIN-RM/2.0E

The 5' flanking region of *HGFIN*, HGFIN-RM/2.0 was as previously reported [12]. The HUGO Gene Nomenclature Committee has suggested the official symbol of HGFIN as GPNMB: human transmembrane glycoprotein nonmetastatic melanoma protein B. A two-step cloning procedure has been used to add exon 1, with the translational start site omitted, downstream of HGFIN-RM/2.0. The clone has been designated HGFIN-RM/2.0E. The first step used PCR with pooled human gDNA as template and Hot Start Ex *Taq *Polymerase (Invitrogen) with the following primers: 5'-ggtgcagggaaggaaaaaagac-3' (sense) and 5'-tagagacattccatgctgaa-3' (antisense). The fragment was inserted into pCR2.1 and was designated HGFIN-RM/2.1. In the next step, we cloned HGFIN-RM/2.0E with primers that include exon 1 with omission of the translation start site: 5'-ctcgaggtgcagggaaggaaa-3' (sense with *Xho*I linker) and 5'-aagctttccatgctgaattcc-3' (antisense with *Hin*dIII linker). The fragment was first ligated into pCR 2.1 for sequencing at the Molecular Core Facility, New Jersey Medical School (Newark, NJ, USA). After the DNA sequencing verification, the insert was subcloned into pGL3-basic reporter vector within *Xho*I/*Hin*dIII sites.

### Transfection and reporter gene assay

Reporter gene assays were performed as described [16]. Briefly, non-tumorigenic cells at 60–80% confluence were co-transfected with pGL3-HGFIN-2.0 or -HGFIN-2.0E and pβ-gal-control (0.2 μg each). Transfections with Effectene (Qiagen, Valencia, CA) results in 60–80% efficiency as determined by labeling for β-gal [16]. Controls were transfected with pGL3-basic pβ-gal. After 48h, cell extracts were quantitated for luciferase and β-gal using kits from Promega and Clontech, respectively. The ratios of luciferase/β-gal in cells transfected with vector alone were normalized to 1. Luciferase activities were presented per μg of total protein and the levels normalized with cells transfected with vector alone. Total protein in cell extracts was quantitated using a protein assay kit from BioRad (Hercules, CA, USA).

### Stable HGFIN knock-out and expression

The method to construct HGFIN-specific siRNA into pPMSKH1 has been described previously [14]. HGFIN siRNA was based on NCBI accession number AF322909 spanning +343/+361: 5'-catttgcggtgaacctgat-3'. Blast analyses using NCBI database determined no significant homology to any human gene. The 19 nucleotide sequence (sense) was placed in tandem with the loop structure followed by the antisense sequence of the upstream 19 nucleotide sequence compliment, resulting in 64 nucleotides. Control siRNA contained single nucleotide mutations within the gene-specific insert. Double-stranded DNA was ligated into pPMSKH1 at a molar ratio of 1:50 (vector to insert). Digestion with *Eco*RI and *Hin*dIII confirmed inserts of ~280 bp. Negative clones without inserts showed bands at ~220 bp. The insert was further verified by DNA sequencing at the Molecular Resource Facility, New Jersey Medical School, Newark, NJ. Stimulated (GM-CSF) bone marrow fibroblasts have been shown to induce HGFIN and were therefore used to verify the efficiency of siRNA in HGFIN knock-out [1].

HGFIN knock-out was performed for MCF12A by co-transfecting with pPMSKH1-HGFIN (wild-type or mutant) and pHyg. Stable transfectants were selected with hygromycin at 5 μg/mL. HGFIN expression was studied by co-transfecting T47D with pFLAG-HGFIN and pHyg. Stable transfectants were selected with 150 μg/mL hygromycin. Selected cells were positive for Flag by western blots with combination of whole cell and membrane extracts (data not shown). The combinations of extracts were necessary as HGFIN is a membrane molecule [1].

### Western blots

Cell membrane extracts were obtained as previously described [17]. Briefly, cells were incubated with 400 μL of 1× lysis buffer (Promega) for 15 min at room temperature. Cell lysates were pelleted by centrifugation at 10,000 *g *for 15 min at 4°C and the membrane fractions were resuspended in 300 μL of PBS and then vortexed. Whole cells extracts were prepared by repeated cycles of freeze–thaw. The membrane and whole cell extracts were combined and then analyzed for total protein using the BioRad protein assay.

Extracts (10 μg total protein) were electrophoresed on 12% SDS-PAGE. Proteins were transferred to PVDF membranes (Perkin Elmer, Wellesley, MA, USA), and then developed with anti-Flag by overnight incubation at 4°C. After this, membranes were washed and incubated with horseradish peroxidase (HRP)-conjugated goat anti-rabbit IgG (1/2,000) for 1 h at 4°C. HRP was developed with chemiluminescence western blot detection reagents (Perkin Elmer). The molecular weights were determined by comparing to Kaleidoscope prestained standards (BioRad).

### Cell migration assay

Cell migration was studied in a transwell system with 8.0 μm inserts using 24-well plates (Falcon, Lincoln Park, NJ, USA). Cells (2 × 10^5^) were re-suspended in sera-free media and then added into the inner chamber to a volume of 500 μL. Plates were incubated at 37°C with 5% CO_2 _for 1.5 h. After this, cells within the inserts were removed with cotton swabs. The filter along with the cells that migrated were fixed and stained with methylene blue. The total numbers of migrated cells were counted with an inverted light microscope (Olympus, Long Island, NY, USA).

### Selection of primary breast cancer cells

Breast tissues were obtained at the initial diagnosis of patients with Stages IIIA or IIIB. At the time of surgery, patients were not subjected to chemotherapy or radiation. The use of breast tissues followed the guidelines of the Institutional Review Board, Newark Campus. Patient 7 was obtained from Cooperative Human Tissue Network, University of Pennsylvania Medical Center (Philadelphia, PA, USA). Variations in the hormone status of patients are summarized in Table 1. Malignant cells within the surgical breast tissues were selected as described previously [18]. The immunohistochemistry analyses were performed with archived samples from the Pathology Department, University Hospital, New Jersey Medical School.

**Table 1:** Breast cancer study subjects and hormone status.

**Subject (patient no. or tissues)**	**Age range (years)**	**ER**	**PR**	**HER2 (IHC)**	***In situ *densities (range)**
1–3	65–73	-	-	-	<0.2–1.2
19–28	54–60				
29–35	36–60				

4–7	55–60	+	+	1+	1–1.5
8–10	60–64				

11–18	70–82	+	+	-	0.5–1
36–50	56–65				

Benign tissues	35–60	Not performed	Not performed	Not performed	10

The demographics of patients and their hormonal status are given. The patients are grouped based on hormonal status and are coded with assigned numbers. All patients were diagnosed with infiltrating ductal carcinomas. ER, estrogen receptor; PR, progesterone receptor; HER2, c-erbB-2; IHC, immunohistochemistry.

### *In situ *hybridization for HGFIN mRNA

Slides with surgical breast samples from benign and malignant subjects were provided blinded by the co-author of this manuscript (MH). Thus, the status of the patients (benign vs malignant) was not revealed until after the results were obtained and analyzed. All patients were diagnosed with infiltrating ductal carcinomas and none with lobular.

*In situ *hybridization was performed with a cocktail of three antisense biotinylated oligonucleotides, 18 nucleotides each, specific for the HGFIN mRNA, as previously described [1,19]. Briefly, the slides were de-waxed and then incubated with 30 μg/mL proteinase K for 1 h at 37°C. Negative control slides were incubated with 100 μg/ml RNase for 30 min at 37°C. After this, slides were prehybridized with 200 ng/mL oligonucleotide cocktail, each with biotin conjugated at the 5' ends. The oligonucleotides were selected from the two ends, and middle regions of HGFIN cDNA, accession number AF322909 [1]: 5'-ccacttgatgccgccaaa-3' (+111/+128); 5'-atggcaccggccaaagcc-3' (+496/+513); 5'-gcctgtggtatgatgtgc-3' (+2235/+2252). Sections were then incubated for 1 h at room temperature with 1.25 μg/ml avidin-AP (Boehringer Mannheim Biochemicals). Control slides were incubated with a cocktail of sense oligomers. Slides were counterstained with Harris Modified Hematoxylin (Fisher Scientific, Springfield, NJ, USA) and then examined microscopically with Olympus AX-70 microscope and a Magnafire digital camera (Olympus) as described previously [20]. Photomicrographs were imported into an image analysis program (analySIS, Soft Imaging System, Munster, Germany) and analyzed to count the positive labelings. Labeling intensities <0.05 were considered negative. The densities of labeling from non-tumorigenic cells were normalized to 10.

### Northern analysis

Northern analysis for steady state HGFIN mRNA was performed as described previously [21]. In brief, total RNA (10 μg) were analyzed with HGFIN cDNA probes, labeled with [α-^32^P]-dATP, 3,000 Ci/mM, (Dupont/NEN, Boston, MA, USA). Membranes were stripped and then re-probed with cDNA for 18S rRNA. Probes were randomly labeled with the Prime-IT II random primer kit (Stratagene, La Jolla, CA, USA). Hybridized membranes were developed in a phosphoimager cassette (Molecular Dynamics, Sunnyvale, CA, USA) and then scanned after 16 h on a PhosphoImager (Molecular Dynamics). cDNA for 18S rRNA was purchased from ATCC.

### Semi-quantitative RT-PCR

Total RNA was extracted from cells and 2 μg was reverse transcribed. cDNA (200 ng) was subjected to PCR for HGFIN using primers designed from accession number AF322909, spanning +570/+681: 5'-aaccttttcctcaccaccc-3' (forward) and 5'-ttcacagaaactctcactgaac-3' (reverse). PCR reactions were normalized by amplifying the same sample of cDNA with primers specific for glyceraldehyde-3-phosphate dehydrogenase (GAPDH). The primers for GAPDH spanned +212/+809 (NM_002046): 5'-ccacccatggcaaattccatggca-3' (forward); 5'-tctagacggcaggtcaggtccacc-3' (reverse). PCR was performed for 35 cycles for HGFIN and 30 cycles for GAPDH at 94°C for 30 s, 55°C for 30 s and 72°C for 30 s with a final extension at 72°C for 10 min. PCR reactions (10 μL) were separated by electrophoresis on 1.0% agarose containing ethidium bromide. Band sizes were compared with 1 kb DNA ladder (Invitrogen).

### Growth curve

Cells were plated at 100 cells/T25 tissue culture flasks. At weekly intervals, cells were trypsinized and then counted.

### Methylcellulose cultures

Clonogenic assays were performed as described previously [14]. Briefly, cells were resuspended in 1.2% methylcellulose containing the respective culture media. Assays were performed with cells seeded at 10^2^/ml in 35 mm suspension dishes. Colonies with >20 cells were counted after 5-day incubation at 37°C.

### Data analyses

Statistical evaluations of the data were performed with analysis of variance and Tukey-Kramer multiple comparisons test. A result of p < 0.05 was considered significant.

## Results

### HGFIN expression in breast cancer cells

*HGFIN *is partly regulated by p53 through multiple binding sites within the 5' flanking region [12]. In addition, *HGFIN *has been linked to cell differentiation and cycling quiescence [1]. The murine homologue of *HGFIN*, *osteoactivin*, has been linked to bone invasion of breast cancer cells [11]. This study further investigated the role of p53 in *HGFIN *expression and its involvement in malignancy of breast cancer.

**Figure 1:**
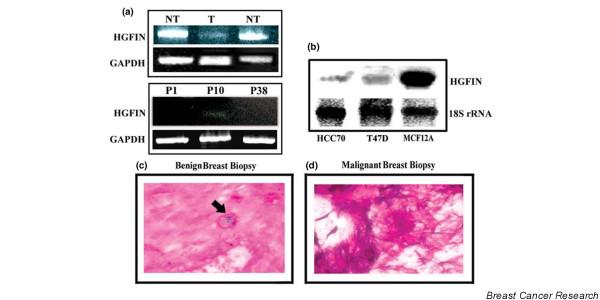
HGFIN expression in breast cells and tissues. **(a) **RT-PCR for HGFIN and GAPDH in non-tumorigenic (NT) (MCF10 and MCF12A) and tumorigenic (T) (DU4475) breast cells. P1, P10 and P38 are the non-identifier codes for patients. **(b) **Total RNA was analyzed by northern analyses for HGFIN from MCF12A non-tumorigenic cells and tumorigenic cell lines (HCC70 and T47D). Membranes were stripped and re-probed for 18S rRNA. **(c) **and **(d) **Representative *in situ *hybridizations for HGFIN from benign breast tissue, *n *= 25 **(c) **and malignant cells, *n *= 50 **(d)**. Arrow shows dense staining for alkaline phosphatase (blue) in the benign section. The images were acquired with 100×/0.3 NA objectives.

We screened paraffin sections of breast biopsies for HGFIN mRNA. This was addressed by *in situ *hybridization with a cocktail of biotin-conjugated oligonucleotides. We also examined malignant breast cells from cell lines and primary sources for HGFIN mRNA. By RT-PCR, we screened two different non-tumorigenic cells (NT), MCF12A and MCF10, which resulted in single bands at the predicted sizes for HGFIN at 112 bp (Figure 1a, top row). In contrast a light band was observed for DU4475 (T) breast cancer cell line (Figure 1a, top row). RT-PCR with selected primary breast cancer cells showed a light band for Stage 0 breast cancer patient (P0) (Figure 1a, lower row, middle lane) and undetectable bands for breast cancer cells selected from Stage III patients (P1 and P38) (Figure 1a, lower row). By northern analyses, we analyzed total RNA from three cell lines; two malignant and one non-tumorigenic. The intensity of banding for MCF12A was >100-fold greater than the malignant cell lines, HCC70 and T47D (Figure 1b).

*In situ *hybridization for HGFIN mRNA analyzed fifty breast biopsies from patients at various stages of breast cancer and benign tissues. Representative labelings showed dense labels for benign sections (Figure 1c, arrow), but undetectable labeling for malignant tissue (Figure 1d). In total, we observed high HGFIN expression in non-tumorigenic breast cells, but reduced expression in malignant breast cancer cells. Table 1 included the hormonal status to show heterogeneity among samples. Despite these differences, the expression of HGFIN depends on malignancy vs benign, irrespective of hormonal status.

### Transformation of HGFIN knock-out MCF12A

**Figure 2:**
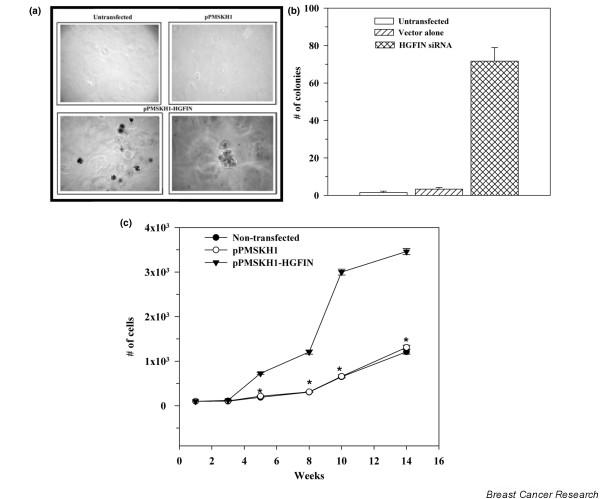
Transformation of HGFIN knock-out MCF12A. **(a) **Representative colonies in 5-day clonogenic assays in methylcellulose with MCF-12A as untransfected, stably transfected with pPMSKH1, or pPMSKH1-HGFIN siRNA. **(b) **The total number of colonies in methylcellulose cultures, plated with 100 cells/dish are presented as mean ± SD, *n *= 5. **(c) **Growth curves were established with MCF-12A as untransfected or stably transfected pPMSKH1 or pPMSKH1-HGFIN siRNA. The total numbers of viable cells were counted at weekly intervals and the results presented as mean ± SD, *n *= 5. *p < 0.05 vs culture with pPMSKH1-HGFIN siRNA.

Malignant cells showed undetectable and reduced expression of *HGFIN *(Figure 1), suggesting a malignant phenotype in cells with reduced *HGFIN *expression. We therefore investigated whether *HGFIN *knock-out in non-tumorigenic MCF12A could confer a transformed phenotype. Knock-out cells were studied for contact-independent growth in methylcellulose matrix and in the growth curve. Control cells were untransfected, stably transfected with vector alone (pPMSKH1) or mutant HGFIN siRNA. Representative dishes for untransfected or vector transfectants showed no colony by day 5 (Figure 2a). HGFIN mutant siRNA showed similar findings (data not shown). HGFIN knock-out MCF12A resulted in large colonies (Figure 2a, lower panels: 100 × left; 400 × right), indicating cell transformation. The total number of colonies with >20 cells were counted and presented as the mean ± SD, *n *= 4. The results showed increased numbers of colonies for HGFIN siRNA cells compared to untransfected and vector transfectants (Figure 2b).

Transformation is generally associated with increased cell growth. We therefore studied the HGFIN knock-out MCF12A in growth curves using 100 cells/dish. The growth curve for vector-transfected MCF12A was similar to untransfected cells (Figure 2c) and mutant HGFIN siRNA transfectants (data not shown). In contrast, HGFIN knock-out MCF12A showed increased cell growth (Figure 2c, triangle symbol). The increases were significant (p < 0.05) as compared to the other experimental points beginning at week 4.

### Reduced clonogenicity in T47D with ectopic HGFIN

**Figure 3:**
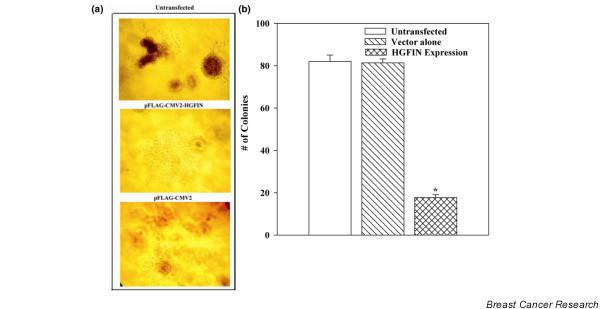
HGFIN imparts contact-dependent growth by T47D. **(a) **Representative (*n *= 5) colonies from clonogenic assays with T47D, as untransfected T47D (top panel); ectopically expressed for HGFIN (middle panel) or transfected with vector alone (lower panel). **(b) **T47D, untransfected or stably transfected with pFLAG-HGFIN were studied in clonogenic assays with 100 cells/35 mm^2 ^dishes. At day 5, the total number of colonies were counted and are presented as mean ± SD, *n *= 5. *p < 0.05 vs untransfected and vector transfectants.

As *HGFIN *knock-out led to increased growth of MCF12A and loss of contact-dependent growth (Figure 2), we next considered whether ectopic expression of HGFIN in a low metastatic cell line could lead to reduced clonogenicity. We stably transfected T47D with pFLAG-HGFIN, and then analyzed growth in methylcellulose cultures. After 5 days, colonies were detected for untransfected cells and vector transfectants (Figure 3, top and lower panels). No colony was observed for HGFIN-expressing T47D (Figure 3, middle panel). The cells did not undergo cell death as determined by trypan blue exclusion (data not shown). We next counted the total number of colonies in cultures plated with 100 cells/dish and observed a significant (p < 0.05) decrease in colonies for HGFIN expressing cells as compared to untransfected T47D and vector transfectants (Figure 3b). In summary, these results show loss of contact independent growth in T47D cells ectopically expressed for HGFIN.

### Effect of HGFIN on the migration of T47D and MCF12A

**Figure 4:**
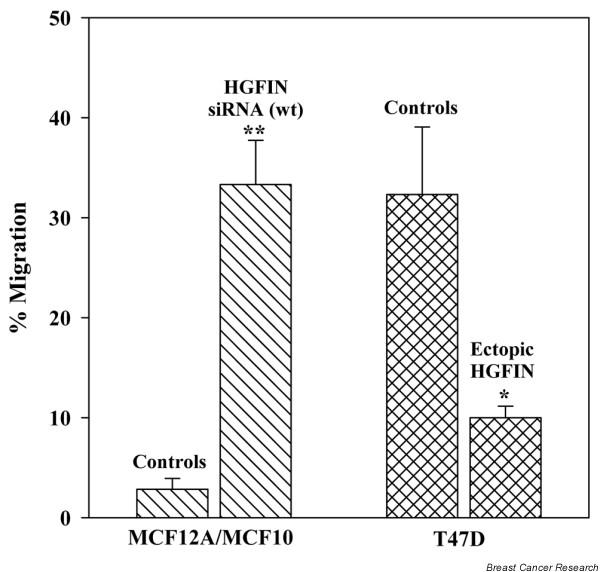
Role of *HGFIN *in cell migration. MCF12A and MCF10 were knock-out for HGFIN, and T47D were ectopically expressed for HGFIN. All cell types were studied in cell migration assays. Controls were performed with MCF12A and MCF10 as untransfected, vector transfectants or transfectants with HGFIN mutant siRNA. Controls for T47D used untransfected cells or vector transfectants. All values for the control groups were pooled and presented together in single bars. The data are presented as mean ± SD, *n *= 5.

As *HGFIN *confers reduced growth rate, and adherent-dependent growth (Figures 2 and 3), we next determined whether these observations correlated with cell migration. Comparisons were made with MCF12A and T47D. MCF12A was stable for HGFIN knock-out and T47D was stably expressed with ectopic pFlag-HGFIN. There was a significant (p < 0.05) increase in HGFIN knock-out MCF12A as compared to untransfected and mutant siRNA transfectants (Figure 4a, left bars). In contrast, ectopic-expression of HGFIN in T47D showed significantly (p < 0.05) reduced migration as compared to untransfected and vector transfectants (Figure 4a, right bars). In summary, HGFIN expression reduced cell migration of T47D and MCF12A.

### Untranslated exon 1 in the activity of *HGFIN *reporter gene

**Figure 5:**
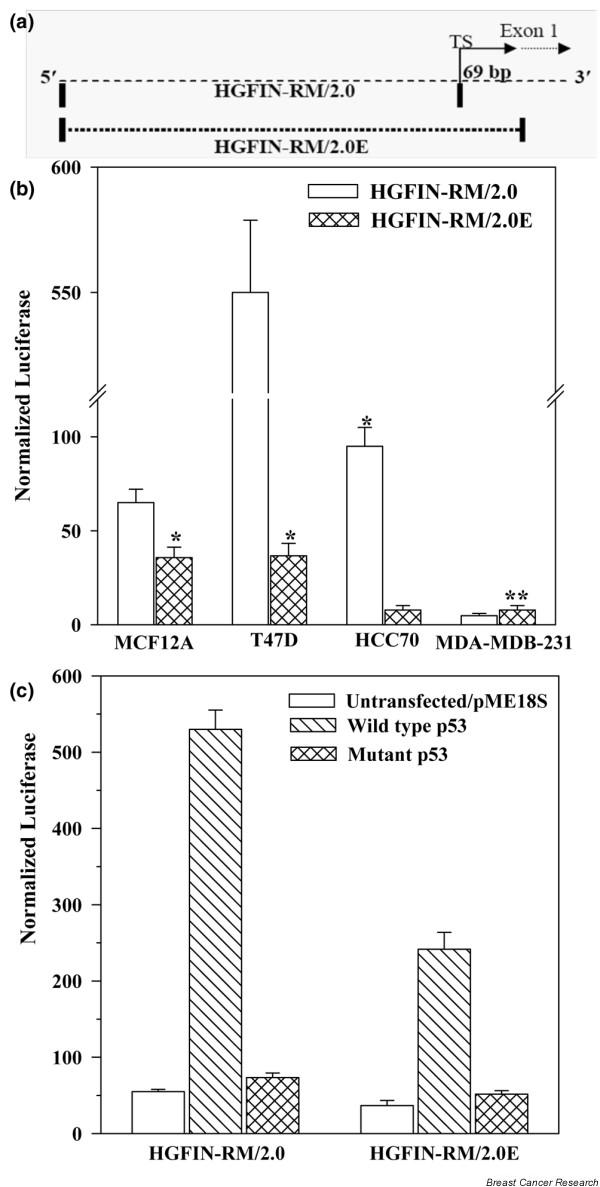
Role of exon 1 in the activity of the 5' flanking region of *HGFIN*. **(a) **Cartoon of the upstream region of *HGFIN*. TS, transcription start site. **(b) **Non-tumorigenic (MCF12A) and tumorigenic (T47D, HCC70, MDA-MB-231) breast cells were co-transfected with pGL3-HGFIN-RM/2.0 or -RM/2.0E and pGal. Luciferase activities were normalized with β-galactosidase activities and the data are presented as the mean ± SD, *n *= 6. *p < 0.05 vs HGFIN-RM/2.0; **p > 0.05 vs HGFIN-RM/2.0E, MDA-MB-231. **(c) **T47D were co-transfected with HGFIN-RM/2.0 or HGFIN-RM/2.0E, and wild-type or mutant p53 expression vectors. Controls were transfected with vector alone. Luciferase activities were determined 16 h after transfection. The data are presented as mean ± SD, *n *= 6.

Previous studies showed an inductive role for p53 in the activity of *HGFIN *by reporter gene activities [12]. The report was based on studies with a 2.0 kb fragment upstream of exon 1, pGL3-HGFIN-RM/2.0. Exon 1 is an untranslated region of HGFIN (Figure 5a). We examined the role of exon 1 in non-tumorigenic cells to determine if this region has regulatory functions. To this end, we used a reporter gene system with exon 1 (minus the translational start site), pGL3-HGFIN-RM/2.0E, to study expressions in MCF12A and three breast cancer cell lines. The studies were compared with pGL3-HGFIN-RM/2.0 in which exon 1 was omitted. HGFIN-RM/2.0E showed a significant (p < 0.05) decrease in luciferase activities in MCF12A, T47D and HCC70 (Figure 5b). There was no significant (p > 0.05) difference between the HGFIN-RM/2.0 and -RM/2.0E in the highly metastatic MDA-MB-231. In addition, luciferase activities were markedly reduced in MDA-MB-231 as compared to the other cell lines. This difference was not due to reduced transfection efficiency as co-transfection with pGal showed β-gal activity similar to the other cell lines (data not shown).

Computer analyses were performed for consensus transcription sites within exon 1 using Genomatix [22]. The output identified a consensus sequence for p53 in exon 1 (Figure 5a). We therefore determined whether high levels of wild-type p53 could reverse the repressive effect of exon 1. This question was addressed with a cell line that expresses low levels of p53, CCL64 [23]. Cells were co-transfected with pGL3-HGFIN-RM/2.0 or -RM/2.0E and/or the following: pME18S-SN3 wild-type p53, pME18S-SCX3 mutant and pPME18S vector alone. As we previously reported that HGFIN-RM/2.0 was under the control of p53, an increase by wild-type p53 was not a surprise (Figure 5c, left bars). Despite ectopic wild-type p53, exon 1 still retained its inhibitory properties, although it was less effective (Figure 5c, right bars). In summary, on these results show a role for wild-type p53 in reversing the suppressive effect of exon 1 in HGFIN activity.

### Role of wild-type p53 in HGFIN reporter gene activity

**Figure 6:**
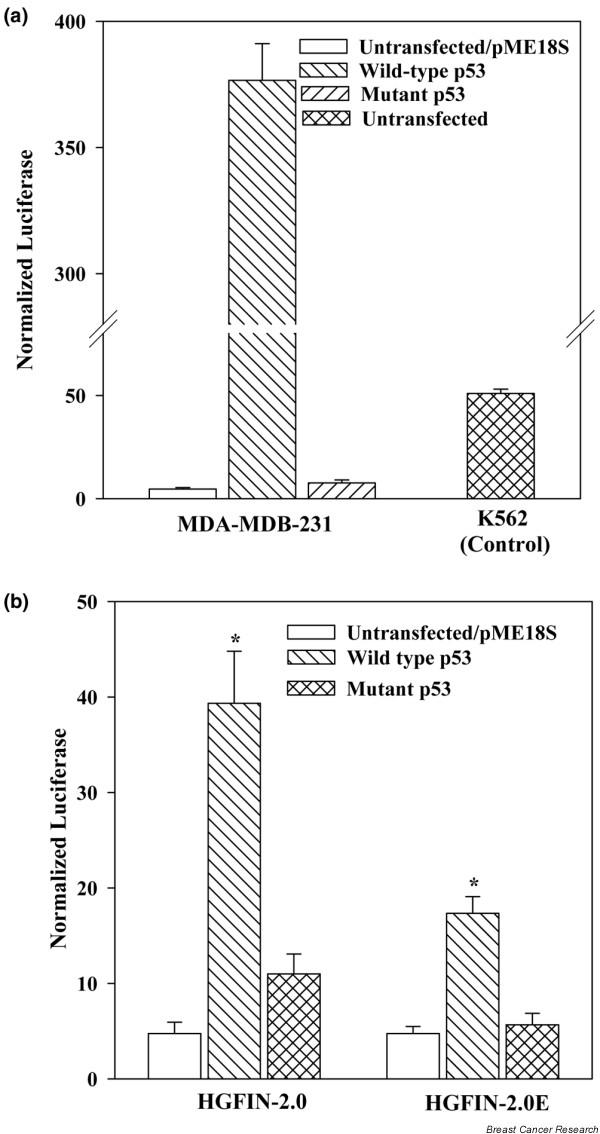
HGFIN reporter gene activity in MDA-MB-231, ectopically expressed for p53. **(a) **MDA-MB-231 was co-transfected with pLuc and/or pME18S-SCX3 expression p53, mutant or vector alone. K562 transfected with pLuc served as control. The results are presented as mean ± SD, *n *= 5. **(b) **MDA-MB-231 was co-expressed with HGFIN-RM/2.0 or HGFIN-RM/2.0E and/or p53 expression vector, pME18S-SCX3. Controls were co-transfected with p53 mutants or vector alone. The results were normalized with β-galactosidase and are presented as mean ± SD, *n *= 6. *p < 0.05 vs vector/untransfected and mutant p53.

MDA-MB-231 expresses mutant p53 [24]. We therefore wanted to verify whether our MDA-MB-231 also expressed functionally mutated p53. This was addressed by transfecting pLuc into MDA-MB-231 with or without ectopic expression of p53 and then determined luciferase activity. Cells were co-transfected with pLuc and/or pME18S-SN3 wild-type p53, pME18S-SCX3 mutant and pPME18S vector alone. Control studies used K562 cells stimulated with 10 nM neurokinin-A that can activate p53 [23]. The result showed significant (p < 0.05) increase in luciferase activity in transfectants with wild-type p53 as compared to vector alone or mutant p53 (Figure 6a). Control studies for pLuc activities were performed with K562, based on previous studies that showed its ability to activate p53 (Figure 6a) [23].

MDA-MB-231 cells were co-transfected with p53 and pGL3-HGFIN-RM/2.0 or -2.0E. In the case of HGFIN-2.0, p53 expression led to increased luciferase by fourfold as compared to p53 mutant transfectants (Figure 6b). A similar increase was not observed for HGFIN-2.0E. This indicates that reduced HGFIN in the highly aggressive MDA-MB-231 could be partly explained by wild-type p53.

## Discussion

This study expands on previous reports that link *HGFIN *and its rodent homolog, *osteoactivin*, to malignancy. We screened primary breast tissues and found *HGFIN *expression in non-tumorigenic cells, but low to undetectable expression in malignant cells (Figure 1; Table 1). The fact that *HGFIN *is regulated by multiple p53 binding sites, combined with other studies linking the human gene to low metastatic potential in melanoma cells suggest that *HGFIN *might function as a tumor suppressor [2,12]. Similar to most genes with tumor suppressor activity, *HGFIN *and *osteoactivin *have also been linked to properties consistent with malignancies [4,11,25]. Our laboratory has just begun to focus on *HGFIN *as a tumor-associated gene, indicating an early investigational stage in studies linked to this gene. To fully understand a role for HGFIN in breast tissues, research studies beyond those presented are required. These include quantitative studies to determine if HGFIN levels are linked to the status of the cancer cells. This could be accomplished in follow-up studies with patients' samples in longitudinal studies, or with isogenic breast cancer cells. It is interesting that *HGFIN *is located on chromosome 7, which is surrounded by microsatellite regions. Thus, it would be of interest to examine malignant cells for loss of the *HGFIN *gene or loss of heterozygosis.

Exon 1 appears to be critical in the control of *HGFIN *expression mainly due to being partly inhibitory in the enhancing function of its activities (Figure 5a,b). It is interesting that the most aggressive cell line, MDA-MB-231 cannot activate *HGFIN *reporter gene activity unless p53 was expressed, suggesting that p53 might be the limiting dysfunction in some cancers with respect to HGFIN expression (Figure 5b,c). In addition to the multiple p53 sites reported for HGFIN-RM/2.0, a consensus sequence has been found in exon 1 [12]. Ectopic expression of p53 led to the activation of HGFIN-RM/2.0E (Figure 5c). However, it is unclear if this increase involves exon 1, as a significant increase was observed for HGFIN-RM/2.0, which has exon 1 omitted (Figure 5c). Exon 1 could be important in unraveling the role of HGFIN in malignancies, not only in breast but also in other cancers. The molecular analysis of HGFIN is the subject of intense research investigation in our laboratory.

We have observed an inverse relationship between *HGFIN *reporter gene activity and the aggressiveness of breast cancer cells (Figure 5b) [26]. The reporter gene activities are consistent with decreased HGFIN mRNA in cancer cells, as compared to non-tumorigenic cells (Figure 1). Although we have shown knock-out of *HGFIN *causes an increase in cell growth, contact independent growth and migration (Figures 2, 3, 4), its role needs to be examined with robust genetic approaches. Indeed, computer analyses have shown evidence of HGFIN within a region of microsatellites, which is linked to instability (data not shown). This observation is currently under investigation, with pairs of autologous samples to show whether loss of *HGFIN *might be an early event in breast cancer transformation.

In summary, the *HGFIN *(or *nmb*) gene and its murine homolog, *osteoactivin *are unexplored in cancer biology and in particular in the capacity of oncogenes. This study has begun the further examination of this gene at the genetic level. Networks comprising HGFIN with cell cycle regulators, established oncogenes and tumor suppressors need to be elucidated. The location of this gene and its control via multiple p53 sites is intriguing, and might have a critical role in tumor biology. The functional behavior of *HGFIN *is reminiscent of the dual role of *p53 *as tumor suppressor and as an oncogene [27,28]. Finally, the hormone status of patients appears to be irrelevant to the functions of HGFIN, suggesting a global function of HGFIN (Table 1).

## Conclusion

*HGFIN *exhibits properties that are consistent with tumor suppressor gene functions. In its absence, non-tumorigenic cells show evidence of transformation and loss of contact dependency as well as increased migration. These findings have been verified with primary breast tissues in which benign tissues show expression of *HGFIN*, whereas malignant tissues shown no evidence of *HGFIN*. The relationship between mutated *p53 *and *HGFIN *expression in malignancy of breast cancer and bone invasion will begin to unravel a new pathway used by p53 in breast cancer biology. Also, as this study was performed by overexpression of one variant of HGFIN, it is unclear how the extra 12 amino acid insert between exons 1 and 2 in the extracellular domain of the other human variant will affect the biology reported in this study [29].

## Competing interests

The authors declare that they have no competing interests.

## Authors' contributions

RLM performed all the assays and prepare a draft of the manuscript; PSP and MB isolated and cultured the primary human breast cancer cells and assisted in preparing the manuscript; MH read the immunohistochemical slides (blinded) and assisted in preparing the manuscript; PR formulated the concept, designed the experiments, and prepared the final manuscript.
